# Geographic variation and factors associated with home-based long-term care utilization across municipalities in Japan

**DOI:** 10.1186/s44263-026-00314-6

**Published:** 2026-09-10

**Authors:** Jennifer Lisa Sakamoto

**Affiliations:** Department of Social Sciences, TU Dortmund University, Dortmund, Germany

**Keywords:** Long-term care, Aging in place, Community-based care, Spatial analysis, Health geography, Regional disparities

## Abstract

**Background:**

Japan’s universal long-term care insurance (LTCI) system promotes aging in place through home-based services (HBS). However, geographic disparities in HBS utilization may persist despite universal coverage. This study examines the following research questions: (1) How does the utilization of HBS vary across Japan? (2) Do HBS utilization rates exhibit spatial clustering or random spatial patterns? (3) What factors are associated with the regional variation in HBS utilization?

**Methods:**

We conducted a cross-sectional study of 1,494 municipalities. Spatial autocorrelation analysis, including Global and Local Moran’s I, was conducted to assess spatial clustering of HBS utilization. Non-spatial and spatial regression models were then estimated to examine regional factors associated with HBS utilization. Based on the Andersen-Newman Model of Health Services Utilization, these factors were categorized as need (older adults certified as having long-term care (LTC) needs), predisposing (sociodemographic characteristics), and enabling factors (municipality’s system resources and LTC service supply). Stratified analyses were conducted to compare results between large urban, small urban, and rural municipalities.

**Results:**

HBS utilization varied widely across municipalities and showed significant spatial autocorrelation. Higher utilization clustered in western Japan, while lower utilization was observed in central and northern regions. The final spatial regression model explained 71.3% of the variance in HBS utilization. The need factor (certified as having LTC needs), predisposing factors (educational attainment), and enabling factors (financial strength of municipality, care manager availability, rurality, institutional care capacity, and non-institutional care provider density) were significantly associated with HBS utilization. Stratified analyses revealed that these associations varied by region type.

**Conclusions:**

Despite universal LTCI coverage, substantial geographic heterogeneity persists in HBS utilization across Japan, associated with underlying municipal characteristics. This variation may pose a challenge to the goal of aging in place. Addressing it will require locally sensitive policy approaches that account for differences in care needs, socioeconomic context, and local LTC service availability across municipalities.

**Supplementary Information:**

The online version contains supplementary material available at https://doi.org/10.1186/s44263-026-00314-6.

## Background

Japan is considered one of the world’s most aged societies, with more than 29.3% of the population being 65 years or older as of 2024 [1]. Among this population, 19.9% of the older adults are certified as needing long-term care (LTC) [2]. In response to the growing demand, Japan implemented its universal long-term care insurance (LTCI) system in 2000, aiming to make the fundamental shift “from care by family to care by society,” and to prevent unnecessary hospitalization of older adults with limitations in activities of daily living through the integration of medical care and welfare services [3, 4].

A key feature of Japan’s LTCI system is its emphasis on supporting aging in place through community-based care rather than institutional care [5]. Within this framework, home-based services (HBS) serve a particularly important role. HBS refers to LTC services that individuals can receive while continuing to live at home and encompasses a wide range of services designed to meet their daily living, medical, and social needs. Under Japan’s LTCI system, HBS includes services such as home care, home nursing, home bathing, short-stay services, and other services, including rental services for welfare equipment [6]. These services enable older adults living with disabilities to remain in familiar environments such as their homes and communities, maintaining their independence, sense of identity, and social connections [7]. From the perspective of healthcare sectors and service providers, HBS is also associated with lower healthcare costs compared to institutional care [8, 9].

Despite the important role of HBS in aging-in-place policies, ensuring its equitable access and utilization remains a challenge. Unlike institutional care, HBS delivery often requires care workers and healthcare providers to travel to service users’ residences, which makes service availability and utilization sensitive to geographic context. A study in the United States identified several barriers hindering access and utilization of home- and community-based services (HCBS) in rural areas, including limited availability of long-term services and supports, inadequate transportation services, telecommunication barriers, threats to business viability, and challenges to caregiving workforce recruitment and retention [10]. Older adults living in rural areas may therefore rely more heavily on informal caregiving, either as a cultural preference or as a response to limited formal care service supply. Similarly, a scoping review found that extended travel distances, travel-related costs, and adverse weather or terrain conditions were also barriers to the provision of hospital-in-the-home services in rural areas [11]. In Japan, a prior study reported comparable demand for home-based medical care in rural and urban areas, yet higher rurality was associated with lower home-based medical care utilization [12]. Another study found that urban areas had more older adults receiving regular home medical visits than rural areas [13]. These findings suggest that geographic context plays a significant role in ensuring the access and utilization of home-based care among older adults.

Although previous studies in Japan have investigated the geographic inequalities in home-based care utilization among older adults, several gaps remain. First, many studies have focused only on medical services, particularly home-based medical or home health care provided by healthcare professionals such as physicians [12–14]. Although these studies offer valuable insights, they capture only one component of care delivered at home. By focusing on HBS, which reflects a broader range of LTC services required to support daily living and aging in place, it may provide a more comprehensive understanding of how community-based care is utilized. Second, limited studies account for spatial dependence when investigating regional disparities and treat municipalities as independent observations. However, there is a possibility that utilization patterns of one municipality may be influenced by neighboring municipalities, as they may share similar LTC coordination networks and regional characteristics. As a result, the geographic inequities in HBS utilization under Japan’s universal LTCI system remain insufficiently understood.

To address these gaps, we conducted a nationwide spatial analysis of HBS utilization across municipalities in Japan. Specifically, we aim to answer the following research questions: (1) How does the utilization of HBS vary across Japan? (2) Do HBS utilization rates exhibit spatial clustering or random spatial patterns? (3) What factors are associated with the regional variation in HBS utilization?

## Methods

### Brief overview of Japan’s LTCI system

Under Japan’s LTCI, all older adults aged 65 years and older (“primary insured persons”), as well as adults aged 40–64 with specified diseases (“secondary insured persons”) can receive LTCI services if they meet the eligibility criteria and are assigned a care level [6]. The LTCI system is financed through a combination of mandatory insurance premiums and general tax revenues from the central and local governments.

To receive LTCI benefits, individuals must undergo a mandatory needs assessment conducted by their local municipality. Based on a standardized functional assessment, a physician’s evaluation, a home-visit report, and deliberation by the LTC approval board, eligible individuals are assigned to one of seven official care levels: support need levels 1–2, for those requiring preventive services, and care need levels 1–5 for those requiring more intensive assistance [3]. Once certified, individuals can receive benefits, including HBS, community-based services (CBS), and institutional-based services, within nationally standardized monthly benefit ceilings that vary by care level. Care planning and service coordination are carried out by certified care managers, who are usually based in care management offices and serve as gatekeepers to the LTCI system. Their responsibilities include assessing care needs, coordinating services from different providers, and monitoring ongoing care [4].

Municipalities play a central role in the planning and delivery of LTCI services, serving as primary insurers of LTCI responsible for care needs assessment, care level certification, care management, and coordination of local service provision [15, 16].

### Study area and data sources

We conducted a cross-sectional study using data from municipalities across Japan. As of 2025, Japan has 1,741 municipalities. The original dataset regarding HBS utilization provided information on 1,573 municipalities, which included individual municipalities and wide-area unions [17]. From this initial dataset, we first excluded 44 municipalities belonging to wide-area unions or lacking the necessary information to calculate HBS utilization. Wide-area unions are joint administrative bodies in which multiple municipalities collectively administer the LTCI system. Because they serve as LTCI insurers rather than individual municipalities, HBS utilization rates cannot be reliably calculated for their member municipalities [18]. These unions are more prevalent in rural areas with smaller populations. Second, we excluded 35 municipalities without geographic neighbors, as spatial analyses require each observation to have defined neighboring units. These were primarily remote or island municipalities located across Japan, such as those in the remote islands of Tokyo, Nagasaki, Kagoshima, and Okinawa prefectures. Following these exclusions, the final sample consisted of 1,494 municipalities.

We obtained data on HBS utilization and care certification levels from the Long-Term Care Insurance Status Report published in December 2024 by the Ministry of Health, Labour and Welfare [17]. Demographic characteristics were obtained from the 2020 Population Census of Japan [19]. Information on system resources and service supply was obtained from the Community-based Integrated Care Basic Data 2025 (Wellness Co., Ltd. Ver. 9.0.2) [20]. The financial strength index of municipalities was obtained from the Major Financial Indicators of Local Governments published in 2023 by the Ministry of Internal Affairs and Communications [21]. Data on population density of the municipalities and average annual income were obtained from Statistical Observations of Municipalities 2022, published by the Statistics Bureau of Japan [22]. Although the data sources span from 2020 to 2025, this reflects the most recent available data at the time of analysis. The dataset and code used in this study are available in the Open Science Framework repository [23].

### Variables

**Fig. 1:**
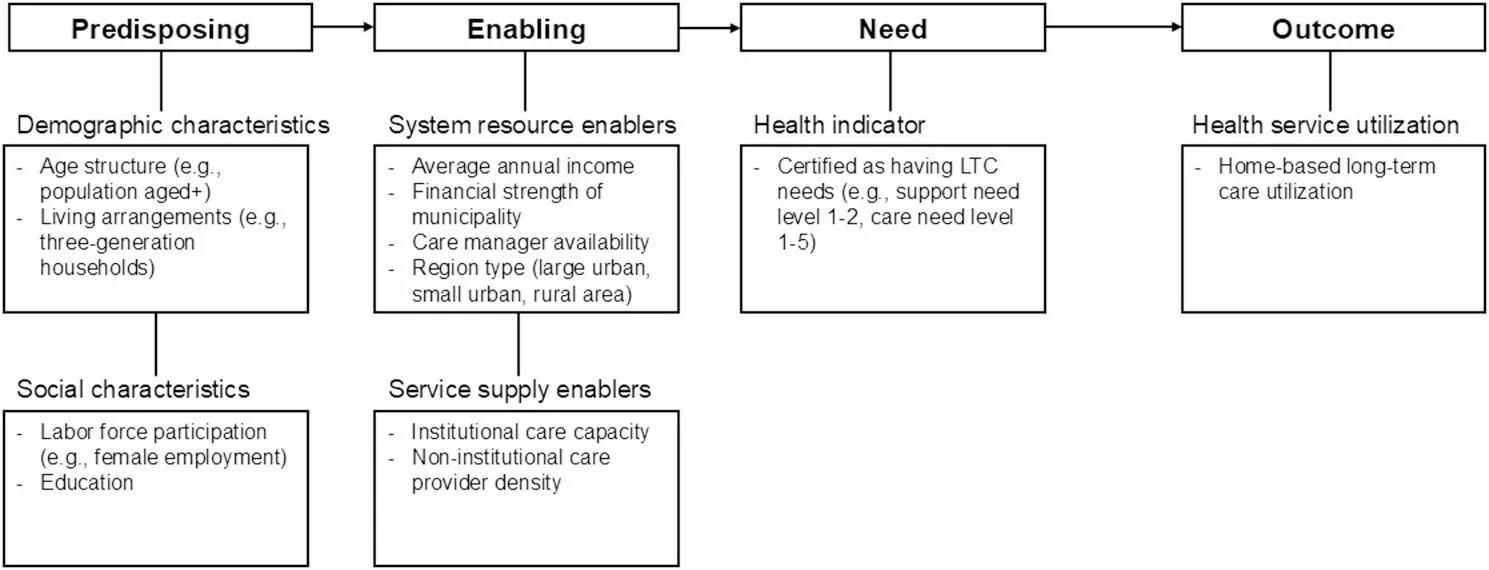
Conceptual framework of regional factors associated with HBS utilization. The framework illustrates the conceptual basis for variable selection in this study, based on the Andersen-Newman Model of Health Services Utilization [25, 26] and a previous Japanese municipal-level study [24]. Determinants of HBS utilization are categorized into need, predisposing, and enabling factors, with enabling factors further divided into system resource enablers and service supply enablers

Figure 1 presents the conceptual framework guiding variable selection, which we developed based on a previous study [24] and Andersen-Newman Model of Health Services Utilization [25, 26]. This framework categorizes determinants of health service utilization into need, predisposing, and enabling factors. Variable definitions and denominators were operationalized following a previous Japanese municipal-level LTC study [24] and based on the availability of municipality-level administrative and census data sources. Supplementary Material 1: Table S1 summarizes the definitions, measurements, denominators, and data sources for all variables included in this study.

### Dependent variable

The outcome variable was the utilization of HBS, which is defined as the share of primary insured persons receiving HBS benefits as of December 2024. Within the Japanese LTCI system, HBS encompasses 12 service types divided into four groups [6]: (1) Home-visit services which includes home-visit care, home-visit bathing, home-visit nursing, home-visit rehabilitation, management guidance for in-home care; (2) Outpatient day-care which includes day care and day rehabilitation; (3) Short-stay services which include short-term stay for care and short-term stay for recuperation; (4) Other services which includes rental or purchase of welfare equipment and designated residential care. Although designed residential care includes residential components, it is administratively classified as HBS rather than institutional nursing home care. It functions as an alternative to permanent institutionalization by offering short-term monitoring and respite care that support aging in place.

### Independent variables

The need factor was represented by the share of older adults certified as having LTC needs (support need levels 1–2, care need levels 1–5).

Predisposing factors included sociodemographic characteristics of the municipality that may influence service utilization. These included the share of residents aged 75 years and older, the share of three-generation households, the share of older adults living alone, employment rates among women and older adults, unemployment rate, and the share of residents with low educational attainment (individuals whose highest educational attainment was elementary or lower secondary school). These factors could influence HBS utilization through their effects on care preferences, availability of informal care, or health literacy.

We divided enabling factors into system resource enablers and service supply enablers to distinguish between different ways in which local conditions may influence HBS utilization. System resource enablers include variables that reflect a municipality’s capacity to plan, coordinate, and support service delivery within the LTCI system, while service supply enablers capture the local availability of organizations that provide direct LTC services. System resource enablers include average annual income, the financial strength index of municipality, the number of care managers per 1,000 primary insured persons, and region type. Average annual income was included as an indicator reflecting the financial resources available within municipalities, as higher municipal income levels may reflect greater financial capacity to support affordability of co-payments and access to formal LTC services. It was calculated by dividing the total taxable income by the number of taxpayers in each municipality, following a previous Japanese study investigating regional disparities using income data [27]. The municipal financial strength index is a measure that captures the municipality’s financial capacity to independently manage and potentially supplement public services, including LTCI provision. It is calculated as the three-year average ratio of the standard fiscal revenue to the standard fiscal need. If the index is 1.0 or higher, it indicates that the municipality can cover its standard expenditure using its own tax revenue, signifying stronger fiscal capacity. This variable serves as a critical measure of the local government’s ability to allocate resources beyond the mandatory national LTCI framework [28]. We classified region type based on population density by habitable land area, as this measure provides a more accurate reflection of the concentration of care resources than administrative municipal labels and has been utilized in previous LTC and healthcare research in Japan [24, 29]. Specifically, municipalities were categorized into three region types: large urban (D > = 1,000 people/km²), small urban (200 < = D < 1,000 people/km²), or rural areas (D < 200 people/km²). This three-category classification was used to balance statistical interpretability with policy relevance and to enable meaningful stratified analyses of how associations vary across urban-rural contexts.

Service supply enablers included two indicators: institutional care capacity per 1,000 primary insured persons and the number of non-institutional care providers per 10,000 primary insured persons. Institutional care capacity was measured as the total capacity of three facility types designated under Japan’s LTCI system: welfare facilities, health facilities, and medical facilities [6]. Other facility-based care services that are not designated under this category, including designated residential care facilities and dementia group homes, were excluded. Non-institutional care providers were defined as all care provider offices delivering direct LTC services that support community dwelling, excluding facility-based care services. This measure aggregates 11 specific care provider categories: home-visit care, home-visit bathing, home-visit nursing, home-visit rehabilitation, day care, day rehabilitation, short stay (care and recuperation), regular visitation for home care, rental service for welfare equipment, and in-home care by a multifunctional small facility. The home-visit care category refers specifically to LTCI-designated home help services provided by certified care workers. General clinics providing home medical visits were not included because they fall under Japan’s medical insurance system rather than the LTCI system. Since this study focuses specifically on HBS utilization within the LTCI framework, supply-side variables were restricted to LTCI-designated providers to ensure conceptual consistency between the dependent and independent variables. Home care support offices, or sometimes translated as care management offices, are also excluded from this measure as they provide care coordination rather than direct LTC services. While these categories include both home-based and community-based administrative classifications, they were combined for this analysis to represent the total regional infrastructure available to support community-dwelling older adults and facilitate aging in place.

### Spatial autocorrelation analysis

We calculated Global and Local Moran’s I to examine whether HBS utilization exhibited spatial clustering patterns. Global Moran’s I measures spatial autocorrelation by examining whether values of a variable at one location are correlated with values at a neighboring location [30, 31]. The value ranges from − 1 to + 1, where positive values indicate spatial clustering of similar values, negative values indicate spatial dispersion, and values close to zero suggest spatial randomness. We used 99,999 permutations to assess statistical significance. Along with Global Moran’s I statistic, we present the Moran scatter plot to visualize the association between HBS utilization in each municipality and its spatially lagged values [32].

Because Global Moran’s I provides only an overall measure of spatial clustering, we calculated Local Moran’s I to identify specific locations of statistically significant clusters and outliers [33]. Based on the Local Moran’s I value and its statistical significance (*p* < 0.05), we classified municipalities as High-High clusters (a statistically significant spatial cluster of high values), High-Low outliers (a statistically significant spatial outlier with a high value surrounded by low values), Low-High outliers (a statistically significant spatial outlier with a low value surrounded by high values), and Low-Low clusters (a statistically significant spatial cluster of low values). We mapped significant spatial clusters and outliers using geographic information systems to examine their spatial distribution.

The spatial relationships between municipalities were defined using a first-order binary contiguity matrix based on the queen criterion, where municipalities were defined as neighbors if they shared either a common border or vertex. We selected this spatial weight matrix because the characteristics of municipalities are likely to be correlated among geographically adjacent areas. The spatial weight matrix was standardized so each row summed to one.

### Non-spatial and spatial regression models

The estimation approach followed a prior study in Germany that investigated the regional variation in the utilization of nursing home care [34]. We first estimated four non-spatial ordinary least squares (OLS) regression models to examine factors associated with HBS utilization across municipalities. Stepwise modeling was used to assess the explanatory power of each domain of variables. Model 1 included need factors only; Model 2 added predisposing factors; Model 3 added system resource enablers; and Model 4 additionally included service supply enablers. We evaluated model fit using adjusted R². To assess the potential for multicollinearity, variance inflation factors (VIF) were calculated. All individual VIF values were below the conventional threshold of 10 (range: 1.19 to 6.19; mean: 2.82), confirming that collinearity was not a significant issue and that the inclusion of all variables was appropriate [35].

To examine whether spatial regression models were necessary, we tested for spatial autocorrelation in the residuals of the full OLS model (Model 4) using Moran’s I statistic. Significant spatial autocorrelation in OLS residuals would indicate violations of the independence assumption and the need for spatial regression models to obtain unbiased and efficient estimates. We then estimated three spatial regression models using maximum likelihood estimation. The spatial lag model (SLM) includes a spatially lagged dependent variable, and it is captured by the spatial autoregressive parameter, ρ (rho). It accounts for potential spillover effects, where HBS utilization in one municipality may be influenced by the utilization in neighboring municipalities. The spatial error model (SEM) accounts for unobserved spatial autocorrelation in the error term (i.e., unobserved factors clustering spatially), captured by the spatial autoregressive parameter, λ (lambda). The spatial autoregressive combined model (SAC) incorporates both spatial spillover (ρ) and spatial error dependence (λ). Model selection was based on Akaike Information Criterion (AIC), with lower values indicating better fit. When AIC values were similar, we utilized the Bayesian Information Criterion (BIC) to determine the preferred specification. This approach prioritizes parsimony, which favors simpler model structures with fewer parameters.

Descriptive statistics and regression analyses were conducted using Stata 18 (StataCorp LLC, College Station, TX). Spatial autocorrelation of HBS utilization was assessed using Global and Local Moran’s I in GeoDa, while spatial autocorrelation in OLS residuals was tested in Stata. Spatial regression models were estimated using the spreg command [36]. Choropleth maps were created using QGIS Desktop version 3.40.14 (QGIS Development Team; QGIS.org, Open Source Geospatial Foundation).

### Sensitivity analyses

To assess the robustness of our findings, we conducted several sensitivity analyses. First, the regression models were re-estimated using an inverse-distance spatial weights matrix instead of the queen contiguity matrix. Second, to test whether the results were sensitive to outliers, we removed municipalities with standardized residuals greater than 2.5 (|z| > 2.5) and compared the results with those from the full sample. Finally, to examine whether factors associated with HBS utilization varied across urban-rural contexts, the final spatial regression model was estimated separately for large urban, small urban, and rural municipalities. For each region type, spatial weight matrices were constructed using queen-based first-order binary contiguity matrix. Municipalities that became isolated after dividing the sample by region type were excluded from the stratified analyses, following the same approach applied in the full sample. This resulted in the exclusion of 31, 22, and 35 municipalities from the large urban, small urban, and rural areas respectively, yielding final sample sizes of 432, 701, and 273 municipalities.

## Results

### Variation in HBS utilization

Table 1 summarizes descriptive statistics for the 1,494 municipalities included in the analysis. The wide ranges observed across all variables indicate substantial geographic variation.

**Table 1:** Descriptive statistics

Variables (unit)	Mean	SD	Min	p25	p50	p75	Max
**Home-based long-term care utilization**							
Older adults receiving HBS (%)	10.6	1.9	2.6	9.3	10.5	11.8	18.9
**Predisposing factors**							
Older adults aged 75 and above (%)	57.4	3.5	45.4	55.3	57.6	59.8	70.6
Three-generation households (%)	7.4	5.0	0.0	3.6	6.2	10.1	29.2
Older adults living alone (%)	14.3	4.7	4.5	10.9	13.4	16.9	35.3
Female employment (%)	47.6	4.7	29.7	44.6	47.7	50.6	70.8
Elderly employment (%)	27.2	5.3	15.6	23.4	26.3	30.0	67.3
Unemployment (%)	3.6	1.0	0.0	3.1	3.6	4.2	9.2
Low educational attainment (%)	19.1	8.8	1.3	12.7	17.9	24.8	53.1
**Enabling factors**							
System resource enablers							
Average annual income (Millions in JPY)	3.1	0.7	2.2	2.8	3.0	3.3	14.0
Financial strength index of municipality	0.5	0.3	0.1	0.3	0.5	0.7	1.9
Care managers (per 1,000 primary insured persons)	2.6	9.4	0.0	2.0	2.5	3.0	12.9
Population density by habitable land (people/km²)	1505.2	2824.9	9.4	237.2	507.3	1308.1	23182.1
Service supply enablers							
Institutional care capacity (per 1,000 primary insured persons)	36.4	22.9	0.0	24.5	32.0	42.9	277.4
Non-institutional care provider density (per 10,000 primary insured persons)	36.5	12.9	0.0	28.8	34.7	42.6	181.8
**Need factor**							
Older adults certified as having LTC needs (%)	18.8	2.9	10.9	16.9	18.7	20.6	35.9

SD standard deviation; Min minimum; Max maximum; p25, p50, p75 25th, 50th, and 75th percentile; HBS home-based service; LTC long-term care; JPY Japanese yen

**Fig. 2:**
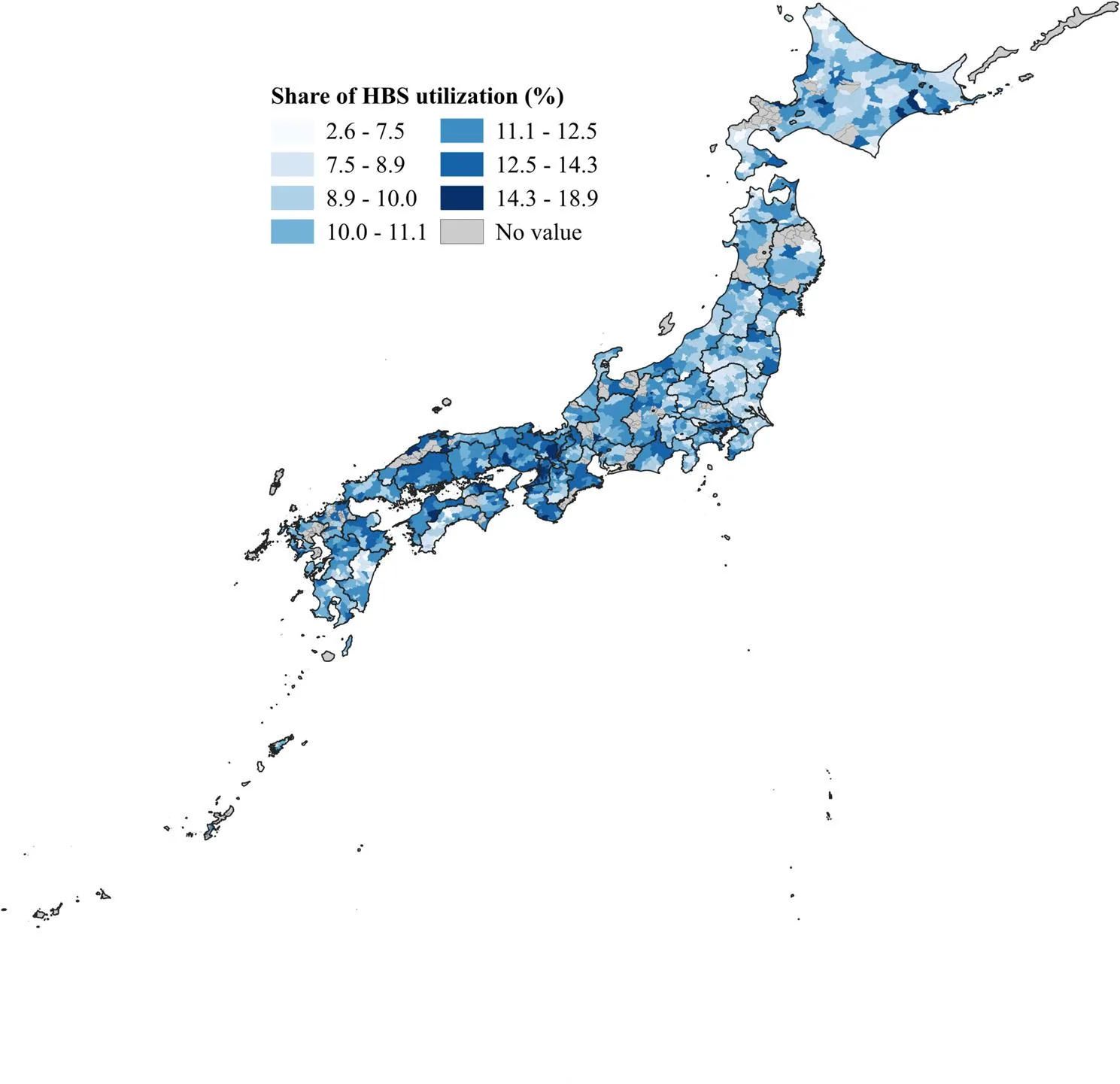
Choropleth map of HBS utilization across municipalities in Japan. The map shows the share of primary insured persons receiving home-based service (HBS) benefits across 1,494 municipalities in Japan as of December 2024. Darker shading indicates a higher share of HBS utilization. Municipalities excluded from the analytical sample (wide-area unions and municipalities without defined geographic neighbors) are shown in grey

HBS utilization varied widely across municipalities, with a mean utilization rate of 10.6% and a range of 2.6% to 18.9%. Specifically, higher utilization rates are concentrated in western Japan, particularly in regions of Kansai and Chugoku, and sporadically throughout Kanto, Tohoku, and Hokkaido. In contrast, lower utilization rates are predominantly observed in Kyushu, Kanto, Tohoku, and Hokkaido regions (Fig. 2).

### Spatial clustering of HBS utilization

**Fig. 3:**
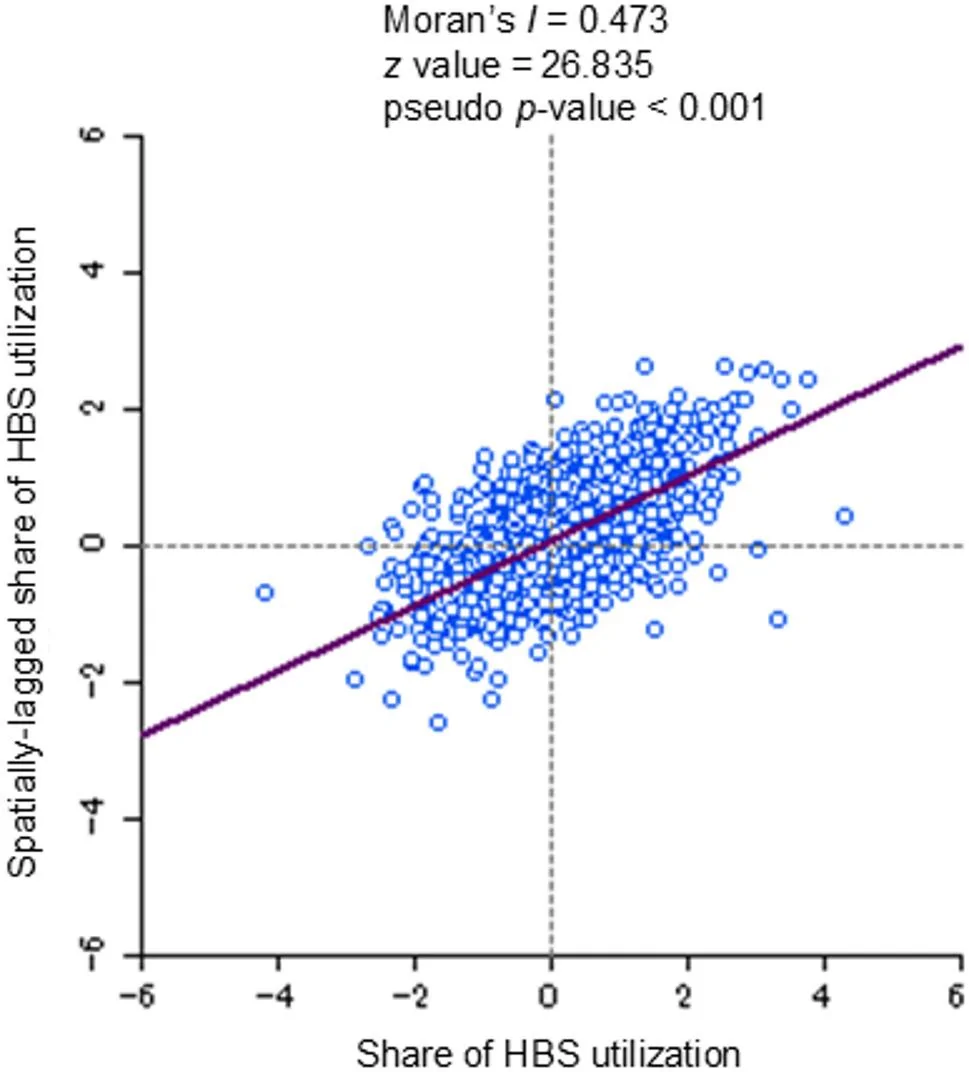
Moran scatter plot of HBS utilization across municipalities. The plot shows the relationship between the standardized share of HBS utilization in each municipality (x-axis) and the spatially lagged standardized value, representing the average utilization among neighboring municipalities (y-axis). The slope of the fitted line corresponds to the Global Moran's I statistic, indicating the overall degree of spatial autocorrelation in HBS utilization

To assess whether the observed patterns reflected spatial clustering rather than random distribution, we examined Global and Local Moran’s I. The Global Moran’s I statistic and corresponding Moran scatter plot indicated statistically significant positive spatial autocorrelation (Global Moran’s *I* = 0.473, *z* value = 26.835, pseudo *p*-value < 0.001), rejecting the null hypothesis of spatial randomness (Fig. 3).

**Fig. 4:**
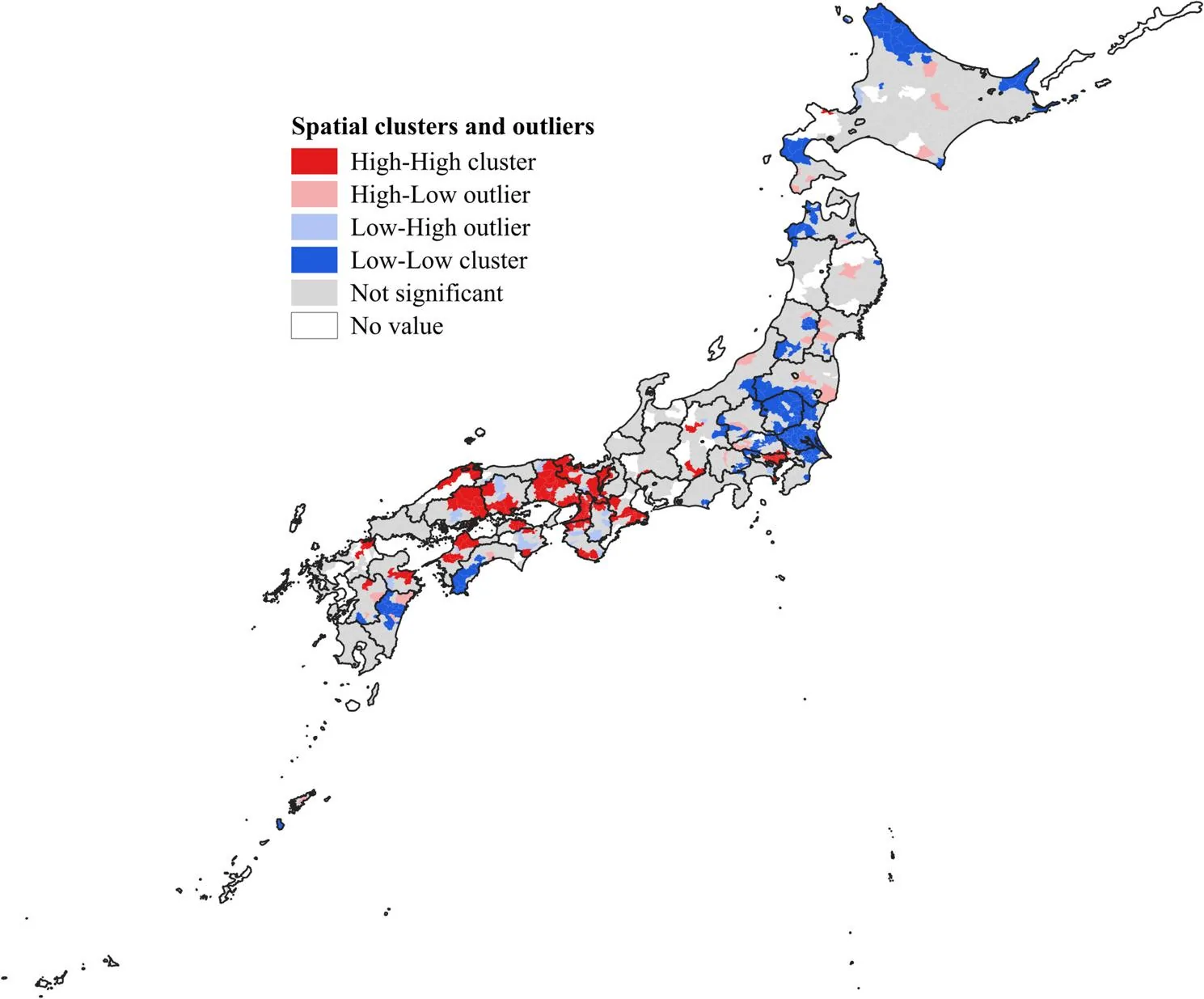
Local Indicators of Spatial Association (LISA) cluster map of HBS utilization across municipalities. The map shows statistically significant (*p* < 0.05) spatial clusters of HBS utilization, based on Local Moran's I statistics calculated using a queen contiguity spatial weights matrix. Cluster types (High-High, High-Low, Low-High, and Low-Low) are indicated in the map key. Municipalities without statistically significant clustering are shown in grey

Furthermore, Fig. 4 presents the Local Indicators of Spatial Association (LISA) cluster map of HBS utilization. The analysis identified the following numbers of significant spatial clusters and outliers at the 5% significance level: 207 High-High clusters, 27 High-Low outliers, 29 Low-High outliers, and 192 Low-Low clusters. High-High clusters, which are municipalities with above-average utilization surrounded by similarly above-average neighbors, were primarily located in western Japan. High-Low outliers, which are municipalities with above-average utilization surrounded by municipalities with below-average utilization, were sporadically observed in Hokkaido, Tohoku, Kanto, and Kyushu regions. In contrast, Low-High outliers, which are below-average utilization surrounded by municipalities with above-average utilization, were mostly located in western Japan, particularly the Kansai, Chugoku, and Shikoku regions. Finally, Low-Low clusters were concentrated in the central and northern areas, including regions of Kanto, Tohoku, and Hokkaido.

### Explaining variation in HBS utilization

Table 2 presents the results of the non-spatial OLS regression models. In the full model, including all control variables, 71.3% of the variation in HBS utilization can be explained. Model 1, which included only the need factor, accounted for 45.5% of the variation. The addition of predisposing factors in Model 2 substantially increased the explanatory power to 64.7%. Incorporating system resource enablers in Model 3 slightly increased the adjusted R² to 69.5%. Finally, the inclusion of service supply enablers in Model 4 achieved an adjusted R² of 71.3%.

**Table 2:** Non-spatial regression results

	Model 1	Model 2	Model 3	Model 4
**Need factor**				
Older adults certified as having LTC needs, share	0.4544*** (0.0507)	0.4999*** (0.0412)	0.4839*** (0.0315)	0.4775*** (0.0289)
**Predisposing factors**				
Older adults aged 75 and above, share		0.0520* (0.0230)	0.0284 (0.0154)	0.0332* (0.0158)
Three-generation households, share		0.0006 (0.0252)	0.0074 (0.0168)	0.0183 (0.0163)
Older adults living alone, share		-0.0233 (0.0275)	0.0038 (0.0240)	-0.0093 (0.0234)
Female employment, share		0.0486* (0.0194)	0.0296 (0.0233)	0.0086 (0.0193)
Elderly employment, share		-0.0130 (0.0215)	0.0131 (0.0179)	0.0176 (0.0177)
Unemployment, share		0.1786* (0.0724)	0.0833 (0.0548)	0.0411 (0.0556)
Low educational attainment, share		-0.0832*** (0.0161)	-0.0556*** (0.0099)	-0.0530*** (0.0092)
**Enabling factors**				
*System resource enablers*				
Average annual income (Millions in JPY)			0.0004 (0.0008)	0.0001 (0.0008)
Financial strength index of municipality			0.0036 (0.0029)	0.0033 (0.0026)
Care managers (per 1,000 primary insured persons)			0.0034*** (0.0004)	0.0027*** (0.0005)
Region type (ref: Large urban area)				
Small urban area			-0.0037*** (0.0010)	-0.0029** (0.0010)
Rural area			-0.0107*** (0.0016)	-0.0096*** (0.0016)
*Service supply enablers*				
Institutional care capacity (per 1,000 primary insured persons)				-0.0001*** (0.0000)
Non-institutional care provider density (per 10,000 primary insured persons)				0.0001* (0.0001)
Constant	0.0207* (0.0085)	-0.0247 (0.0184)	-0.0201 (0.0181)	-0.0087 (0.0176)
Observations	1494	1494	1494	1494
adj. R²	0.455	0.647	0.695	0.713

LTC long-term care; JPY Japanese yen. *p < 0.05; **p < 0.01; ***p < 0.001; Standard errors clustered at the prefecture level are presented in parentheses.

### Comparison of spatial model specifications

Spatial autocorrelation in the residuals of the full OLS model (Model 4) was tested to examine whether spatial regression models were necessary. The Moran’s I test statistic followed a chi-square (χ²) distribution and revealed significant positive spatial autocorrelation in the residuals (χ² = 175.63, *p* < 0.001), indicating violations of the independence assumption and justifying the need for spatial regression modeling.

We estimated three spatial regression models to account for spatial dependence: SLM, SEM, and SAC. Supplementary Material 1: Table S2 presents the model comparisons across all models with their respective AIC and BIC values. The SEM and SAC models outperformed the OLS and SLM based on the AIC. Although their AIC values were nearly identical (∆*AIC* = 0.698), the SEM was selected as the preferred model based on its lower BIC (∆*BIC* = 6.007) and the principle of parsimony. Furthermore, the SAC model’s spatial lag parameter was not statistically significant (ρ = 0.043, *p* > 0.05), while the spatial error parameter remained significant in both the SAC (λ = 0.410, *p* < 0.001) and the SEM (λ = 0.451, *p* < 0.001).

### Factors associated with HBS utilization

Table 3 presents the comparison between the non-spatial OLS model (Model 4) and the selected SEM. The need factor maintained the strongest positive association with HBS utilization in both models (OLS: β = 0.478, *p* < 0.001; SEM: β = 0.476, *p* < 0.001). Among predisposing factors, low educational attainment showed a significant negative association in both models (OLS: β = −0.053, *p* < 0.001; SEM: β = −0.053, *p* < 0.001). Some variables showed different significance levels between the two models. The share of older adults aged 75 and above was positively associated with utilization in the OLS model (β = 0.033, *p* < 0.05) but not statistically significant in the SEM. Conversely, three-generation households (β = 0.023, *p* < 0.05) and unemployment rate (β = 0.083, *p* < 0.05) became statistically significant in the SEM.

Regarding system resource enablers, care manager availability showed consistent positive associations across both models (OLS: β = 0.003, *p* < 0.001; SEM: β = 0.002, *p* < 0.001). Financial strength index of the municipality was positively associated with utilization only in the SEM (SEM: β = 0.004, *p* < 0.05). Rurality was negatively associated with HBS utilization in both models, with small urban areas (OLS: β = −0.003, *p* < 0.01; SEM: β = −0.002, *p* < 0.01) and rural areas (OLS: β = −0.010, *p* < 0.001; SEM: β = −0.007, *p* < 0.001) showing lower utilization compared to large urban areas. Average annual income was not associated with utilization in either model. Among service supply enablers, institutional care capacity showed negative associations (OLS: β = −0.0001, *p* < 0.001; SEM: β = −0.0001, *p* < 0.001), while non-institutional care provider density showed positive association (OLS: β = 0.0001, *p* < 0.05; SEM: β = 0.0001, *p* < 0.001) in both models.

**Table 3:** Regression results of non-spatial model and SEM

	Model 4	SEM
**Need factor**		
Older adults certified as having LTC needs, share	0.4775*** (0.0289)	0.4761*** (0.0129)
**Predisposing factors**		
Older adults aged 75 and above, share	0.0332* (0.0158)	0.0157 (0.0115)
Three-generation households, share	0.0183 (0.0163)	0.0233* (0.0098)
Older adults living alone, share	-0.0093 (0.0234)	0.0001 (0.0148)
Female employment, share	0.0086 (0.0193)	0.0009 (0.0128)
Elderly employment, share	0.0176 (0.0177)	0.0042 (0.0107)
Unemployment, share	0.0411 (0.0556)	0.0831* (0.0348)
Low educational attainment, share	-0.0530*** (0.0092)	-0.0533*** (0.0062)
**Enabling factors**		
*System resource enablers*		
Average annual income (Millions in JPY)	0.0001 (0.0008)	0.0004 (0.0006)
Financial strength index of municipality	0.0033 (0.0026)	0.0039* (0.0018)
Care managers (per 1,000 primary insured persons)	0.0027*** (0.0005)	0.0020*** (0.0003)
Region type (ref: Large urban area)		
Small urban area	-0.0029** (0.0010)	-0.0024** (0.0009)
Rural area	-0.0096*** (0.0016)	-0.0071*** (0.0013)
*Service supply enablers*		
Institutional care capacity (per 1,000 primary insured persons)	-0.0001*** (0.0000)	-0.0001*** (0.0000)
Non-institutional care provider density (per 10,000 primary insured persons)	0.0001* (0.0001)	0.0001*** (0.0000)
Constant	-0.0087 (0.0176)	0.0049 (0.0095)
Observations	1494	1494
Spatial parameter (λ)	-	0.4514*** (0.0293)

LTC long-term care; JPY Japanese yen; OLS ordinary least squares; SEM spatial error model; λ spatial autoregressive (error) parameter. *p < 0.05; **p < 0.01; ***p < 0.001; OLS uses standard errors clustered at the prefecture level. SEM uses robust standard errors

### Sensitivity analyses

When re-estimating the SEM model using an inverse-distance spatial weights matrix, spatial error parameter was larger with inverse distance weights (SEM: λ = 2.566), however, coefficient estimates and significance levels remained consistent. In the outlier sensitivity analysis, a total of 29 municipalities were removed from the analysis, and model fit improved from an adjusted R² of 71.3% in the full sample to 78.2% in the restricted sample, while the main associations remained similar.

Table 4 presents stratified analyses by region type. The need factor remained the strongest predictor of HBS utilization but declined gradually from large urban (β = 0.553, *p* < 0.001) to small urban (β = 0.487, *p* < 0.001) to rural areas (β = 0.414, *p* < 0.001). Low educational attainment was negatively associated with HBS utilization in small urban (β = *−*0.045, *p* < 0.001) and rural areas (β = *−*0.056, *p* < 0.001), but not in large urban areas. Several predisposing factors showed inconsistent associations across region types. Average annual income was not significantly associated with HBS utilization in any region type and the financial strength of the municipality was significant only in rural areas (β = 0.013, *p* < 0.05). Care manager availability showed positive associations, while institutional care capacity maintained negative associations in all regions. Non-institutional care provider density showed positive associations in large urban (β = 0.0002, *p* < 0.001) and small urban areas (β = 0.0001, *p* < 0.05) but not in rural areas. Furthermore, the spatial error parameter was significant in all region types.

**Table 4:** Final regression model estimated by region type (large urban, small urban, and rural area)

	SEM	SEM Large urban	SEM Small urban	SEM Rural
**Need factor**				
Older adults certified as having LTC needs, share	0.4761*** (0.0129)	0.5534*** (0.0194)	0.4874*** (0.0177)	0.4143*** (0.0330)
**Predisposing factors**				
Older adults aged 75 and above, share	0.0157 (0.0115)	0.0108 (0.0159)	0.0583*** (0.0166)	-0.0269 (0.0297)
Three-generation households, share	0.0233* (0.0098)	-0.0043 (0.0288)	-0.0065 (0.0139)	0.0190 (0.0209)
Older adults living alone, share	0.0001 (0.0148)	0.0195 (0.0227)	-0.0310 (0.0225)	0.0020 (0.0365)
Female employment, share	0.0009 (0.0128)	0.0354* (0.0174)	0.0217 (0.0199)	-0.0173 (0.0320)
Elderly employment, share	0.0042 (0.0107)	0.0086 (0.0185)	-0.0172 (0.0149)	0.0139 (0.0261)
Unemployment, share	0.0831* (0.0348)	0.0710 (0.0611)	-0.0481 (0.0477)	0.1255 (0.0918)
Low educational attainment, share	-0.0533*** (0.0062)	-0.0207 (0.0167)	-0.0446*** (0.0085)	-0.0556*** (0.0151)
**Enabling factors**				
*System resource enablers*				
Average annual income (Millions in JPY)	0.0004 (0.0006)	0.0011 (0.0006)	0.0029 (0.0019)	-0.0019 (0.0018)
Financial strength index of municipality	0.0039* (0.0018)	0.0036 (0.0022)	-0.0024 (0.0026)	0.0131* (0.0063)
Care managers (per 1,000 primary insured persons)	0.0020*** (0.0003)	0.0016** (0.0006)	0.0019*** (0.0004)	0.0016* (0.0007)
Region type (ref: Large urban area)				
Small urban area	-0.0024** (0.0009)	-	-	-
Rural area	-0.0071*** (0.0013)	-	-	-
*Service supply enablers*				
Institutional care capacity (per 1,000 primary insured persons)	-0.0001*** (0.0000)	-0.0001*** (0.0000)	-0.0001*** (0.0000)	-0.0001*** (0.0000)
Non-institutional care provider density (per 10,000 primary insured persons)	0.0001*** (0.0000)	0.0002*** (0.0000)	0.0001* (0.0000)	0.0001 (0.0001)
Constant	0.0049 (0.0095)	-0.0312* (0.0131)	-0.0201 (0.0153)	0.0446 (0.0233)
Observations	1494	432	701	273
Spatial parameter (λ)	0.4514*** (0.0293)	0.4095*** (0.0469)	0.3485*** (0.0376)	0.2471*** (0.0590)

LTC long-term care; SEM spatial error model; JPY Japanese yen; λ spatial autoregressive (error) parameter. *p < 0.05; **p < 0.01; ***p < 0.001; For each region type, spatial weight matrices were constructed using queen-based first-order binary contiguity matrix. Robust standard errors are presented in parentheses

## Discussion

This study examined regional variation in HBS utilization across municipalities in Japan and identified several factors associated with these patterns. Despite Japan’s universal LTCI framework, HBS utilization ranged from 2.6% to 18.9% across municipalities and showed substantial geographic heterogeneity with significant spatial clustering. The observed regional characteristics explained 71.3% of the variation in HBS utilization, indicating that much of the geographic disparity can be attributed to differences in need, predisposing, and enabling factors. Although the spatial autocorrelation analysis shows that HBS utilization is geographically clustered rather than randomly distributed, results from non-spatial and spatial regression analyses were similar and key coefficients remained largely consistent across both models. This suggests that while spatial dependence is present, the associations between regional characteristics and HBS utilization are not primarily explained by spatial dependence. Nevertheless, the significant spatial error parameter across all region types indicates that neighboring municipalities tend to share characteristics beyond those captured by the observed variables and unobserved spatial correlated factors continue to play a role in shaping patterns of HBS utilization at the municipality level.

The need factor, represented by the share of older adults certified as having LTC needs, was positively associated with HBS utilization across all models. These findings are consistent with prior LTC research, which shows that impairments in daily activities due to disability are associated with increased use of LTC services [37–40]. However, the magnitude of this association declined across the urban-rural gradient, suggesting that similar levels of care need may translate less consistently into formal service use in rural areas. This may be due to differences in LTC service availability and delivery capacity, as well as greater reliance on informal care pathways in rural settings [41].

Predisposing factors also contributed to variation in HBS utilization. Low educational attainment was negatively associated with utilization across all models, with stratified analyses showing that this association was evident in small urban and rural municipalities. This finding may reflect several mechanisms, including reduced health literacy, financial barriers, or broader socioeconomic disadvantages. Although average annual income was included as an enabling factor to help disentangle these mechanisms, it was not significantly associated with HBS utilization in any region type in the stratified analyses, suggesting that municipality-level income alone does not fully explain the educational attainment pattern. Instead, educational attainment at the municipality level may serve as a proxy for concentrated socioeconomic disadvantages. Lower health literacy may limit awareness of available LTCI services and the ability to navigate the system, while weaker community social capital may reduce access to information about services through informal networks. Furthermore, municipalities with higher shares of residents with low educational attainment may also have greater reliance on informal care networks that substitute for formal service use. Fully disentangling roles of health literacy, social capital and individual-level socioeconomic status requires further research beyond the scope of the current study. Other predisposing factors, including the share of older adults aged 75 and above, three-generation households, female employment, and unemployment rate showed inconsistent associations across models and region types. This suggests that once need and enabling factors are accounted for, these demographic characteristics appear to contribute less to municipality-level variation, although they may remain as important factors in individual-level decisions on service utilization.

System resource enablers also showed important associations with HBS utilization. We found that care manager availability was positively associated across all models and region types, showing their important gatekeeping role in facilitating access and utilization of LTCI services [42]. The financial strength of the municipality was positively associated with HBS utilization in the overall SEM and stratified analysis suggest that this association was only significant in rural municipalities. This may suggest that fiscal resources are more strongly related to HBS utilization in these settings, where supplemental funding may be needed to overcome structural barriers to service delivery, such as low population density and provider recruitment challenges [43]. Additionally, region type remained associated with utilization across all models, with rural municipalities exhibiting lower HBS utilization than large urban municipalities. This pattern is consistent with previous studies documenting urban-rural differences in the use of formal care [41, 44, 45] and highlights the persistence of geographic disparities in LTC service utilization under a universal insurance framework.

Among service-supply enablers, a higher density of non-institutional care provider density was positively associated with HBS utilization in the overall model and in large urban and small urban areas in stratified analyses, suggesting that greater local service supply facilitates the use of HBS in more urbanized settings. The association was not significant in rural areas, which may reflect geographic barriers to service access or greater reliance on informal and institutional care pathways in these settings, despite the presence of providers. Institutional care capacity was negatively associated with HBS utilization across all models and region types. This finding is consistent with prior research suggesting a degree of substitution between facility-based and home-based care [46]. However, the direction of this association should be interpreted with caution. In some municipalities, institution-based care may have expanded before Japan’s current policy emphasis on aging in place and created care pathways that continue to influence how LTC services are currently used. In addition, municipalities where HBS delivery is logistically challenging due to geographic constraints or workforce shortages may have developed greater institutional care capacity as a more practical and reliable alternative to home-based care.

Taken together, these findings suggest that geographic variation in HBS utilization under Japan’s universal LTCI system reflects a complex interplay of need, socioeconomic, and structural factors that operate differently across urban and rural contexts. While the LTCI system appears to be functioning in that need is the strongest predictor of utilization, the persistent urban-rural gradient across multiple factors, including educational attainment, fiscal capacity, and service supply, suggests that universal coverage alone is insufficient to ensure uniform utilization across municipalities. These findings point to the importance of locally sensitive approaches to LTC policy that go beyond insurance coverage to address the underlying social and structural factors associated with LTC service utilization.

The present study is subject to several limitations. First, the cross-sectional design limits our ability to draw causal conclusions, and the observed associations should not be interpreted as directional effects. For example, we found that greater care manager availability was associated with higher HBS utilization. However, we cannot determine whether care managers increase service use or whether municipalities with higher HBS utilization employ more care managers in response to demand. Second, the administrative data used in this analysis does not capture other important dimensions of care, such as service quality, unmet needs, or individual-level factors, including individual care preferences, caregiving burden within households, or perceptions of formal care. In particular, regional variations in cultural norms around family care obligations, including traditional expectations of filial care in Japan, may influence the extent to which families seek formal HBS or rely on informal caregiving. Third, this study focused specifically on HBS rather than the broader category of HCBS. Although the inclusion of CBS was initially considered, their utilization rate was substantially lower (mean = 2.5%) and exhibited different geographic patterns, resulting in poor model fit (adjusted R² = 0.223). For this reason, HBS and CBS utilization were not combined in the analysis. Fourth, as an ecological study using municipality-level data, our findings describe associations at the population level and should not be interpreted as individual-level associations. Finally, both the HBS utilization measure and non-institutional care provider density measure rely on aggregated data. The HBS utilization measure aggregates 12 service categories as classified by Japan’s LTCI system. While this approach is appropriate for a comprehensive geographic overview of HBS utilization, it limits the understanding of association patterns unique to specific service types. The non-institutional care provider density measure aggregates 11 provider categories derived from the Long-Term Care Service Disclosure System via a compiled dataset. As providers are registered separately for each service category they are licensed to deliver, this measure may involve a degree of double counting if a single provider organization holds licenses for multiple service categories, potentially inflating provider density estimates in municipalities where providers tend to offer multiple services.

Future research could address the limitations through complementary approaches. Multilevel analyses incorporating both individual-level and municipality-level data could examine whether older adults with similar care needs receive equivalent services regardless of where they live and provide further understanding on individual and contextual factors that are associated with or contribute to HBS utilization. Service-specific analyses incorporating more granular utilization data and supply indicators would provide deeper insights into the mechanisms underly the geographic patterns identified in this study. Furthermore, qualitative approaches could further shed light on the barriers to service navigation, the role of cultural norms around family caregiving, and logistical challenges in rural areas that are not fully captured by administrative data.

## Conclusions

This study examined geographic variation in HBS utilization across municipalities in Japan and its association with regional characteristics. First, HBS utilization varied substantially across municipalities, ranging from 2.6% to 18.9%. Second, this variation was not randomly distributed but exhibited significant spatial clustering, with high utilization concentrated in western Japan and low utilization in central and northern regions. Third, applying the Andersen-Newman Model of Health Services Utilization, we explained 71.3% of this variation, identifying need, predisposing, and enabling factors associated with HBS utilization. Stratified analyses further revealed that these associations vary across urban and rural contexts. Together, these findings suggest that universal LTC coverage alone does not ensure geographically equitable utilization of HBS, and that regional characteristics were associated with geographic variation in HBS utilization, which may challenge the goal of supporting aging in place. Addressing these patterns will require locally sensitive policy approaches that account for differences in care needs, socioeconomic context, and LTC service availability across municipalities. 

## Supplementary Information

Below is the link to the electronic supplementary material.

Supplementary Material 1: Table S1. Description of variables and sources, aggregated at municipal level (description of variables, denominators, and data sources). Table S2. Comparison of non-spatial and spatial regression models (comparison of model fit statistics of non-spatial and spatial regression models)

## Data Availability

The data analyzed in this study were obtained from publicly available sources, including the Ministry of Health, Labour and Welfare [17], Wellness Co., Ltd. [20], the Ministry of Internal Affairs and Communications [21], and the Statistics Bureau of Japan [19, 22]. The dataset and code generated during this study are available in the Open Science Framework repository: https://doi.org/10.17605/OSF.IO/CE8SM [23].

## Abbreviations

AIC: Akaike Information Criterion
BIC: Bayesian Information Criterion
HBS: Home-based services
HCBS: Home- and community-based services
LISA: Local Indicators of Spatial Association
LTC: Long-term care
LTCI: Long-term care insurance
OLS: Ordinary least squares
SAC: Spatial autoregressive combined model
SEM: Spatial error model
SLM: Spatial lag model
VIF: Variance inflation factor
